# Integrating a quality improvement experiential platform into medical student education

**DOI:** 10.1186/s12909-025-06709-7

**Published:** 2025-02-06

**Authors:** Amy S. Stanley, René M. Kronlage, Miranda J. Reid, Hannah G. Rains, Colleen J. Kalynych, Michele N. Lossius, Janice A. Taylor, Carolyn K. Holland

**Affiliations:** 1 University of Florida College of Medicine, Gainesville, FL USA; 2 University of Florida College of Medicine - Jacksonville Office of Educational Affairs, Jacksonville, FL USA; 3 Department of Pediatrics, University of Florida College of Medicine, Gainesville, FL USA; 4 Department of Surgery, Division of Pediatric Surgery, University of Florida College of Medicine, Gainesville, FL USA; 5 Department of Emergency Medicine, Division of Pediatric Emergency Medicine, University of Florida College of Medicine, Gainesville, FL USA

**Keywords:** Quality improvement, Medical education, Patient safety, Equal access clinic, Free clinic

## Abstract

**Background:**

The call for quality improvement and patient safety (QI/PS) education has increased at every level of medical education. Here, the authors present a QI/PS experiential platform implemented at the University of Florida College of Medicine (UFCOM). The project established a peer-taught hands-on learning platform in a student-run clinic allowing participants to learn and apply QI/PS concepts and tools in a real-world clinic environment. The aims were to assess students’ perceptions in regard to (1) student confidence in quality improvement (QI) methodology, and (2) competency in executing QI initiatives in healthcare as measured by a post-participation survey.

**Method:**

A medical student-led quality improvement team was embedded within University of Florida’s (UF’s) existing student-run clinic network. The QI/PS student-team collaborated with clinic leaders and utilized QI/PS tools to establish, monitor, and expand impactful Plan-Do-Study-Act (PDSA) cycles. The impact of the training was then evaluated using The New World Kirkpatrick Model leveraging a post-project survey which included the Beliefs, Attitudes, Skills, and Confidence in Quality Improvement (BASiC-QI) survey and questions on overall student perceptions.

**Results:**

This project demonstrated positive results in all four levels of Kirkpatrick evaluation: (1) Reaction, (2) Learning, (3) Behavior, and (4) Results. This was shown through (1) a voluntary feedback survey that reported positive feedback from participants with 93% of respondents indicating they “strongly agreed” or “agreed” to positive perception questions; (2) significantly higher scores (*p* < 0.001) on the BASiC-QI Scale for project participants vs. non-participants; (3) the completion of 4.25 PDSA cycles per QI team; and (4) a 10.1% reduction in median patient time in clinic.

**Conclusions:**

This study supports the utility of incorporating a student-led QI/PS interactive platform into student-run clinics to increase knowledge and attitude in implementing QI/PS endeavors while simultaneously improving clinic metrics and outcomes for patients.

## Introduction

Quality improvement and patient safety (QI/PS) education measures have been identified as Common Program Requirements of the Accreditation Council for Graduate Medical Education (ACGME) in the United States and are required competencies by the Canadian Medical Education Directive for Specialists (CanMEDS) Competency Framework [1, 2]. The Association of American Medical Colleges (AAMC) has also placed an increased emphasis on QI/PS concepts being taught throughout the preclinical medical education curricula and has provided competencies and benchmarks for colleges of medicine to use [3–8]. Domain II: Quality Improvement of the *AAMC Quality Improvement and Patient Safety Competencies Across the Learning Continuum* emphasizes recent medical school graduates entering residency should have (1) competency participating in a local quality improvement initiative; (2) demonstrate knowledge of basic quality improvement (QI) methodologies and quality measures; (3) be able to describe basic principles and approaches for creating and sustaining change in QI; and (4) be able to describe strengths, weaknesses, and appropriate uses of measurement and analytic approaches relevant to QI (i.e. run charts, process-control charts); among others [2]. As medical schools work to incorporate longitudinal QI/PS training within the curricula, rarely are there opportunities for students to apply QI knowledge and principles.

Many U.S. medical schools have established free clinics in which student volunteers work to gain medical skills under the mentorship of faculty [9, 10]. A 2014 study found that of the 81.1% of respondents from U.S. AAMC member institutions, there were 106 student-run clinics. This same study also showed over half the student population (57.8%) were involved in these clinics throughout their four years of medical education training [10]. 

There is a growing body of research demonstrating the benefits of peer- and near-peer teaching in medical education, for both the peer teacher and the recipient. Recipients of peer- and near-peer teaching benefit from “cognitive and social congruence” through a shared knowledge base and communication of concepts at a more learner-friendly level. Moreover, learners may feel more at ease than when taught by faculty or senior clinicians [11]. Peer teachers benefit by being better-prepared to communicate and educate as a resident and future attending.

The *National Training Laboratories Institute of Applied Behavioral Science Learning Pyramid* highlights how learners retain more from opportunities to apply their learning and additional studies support the finding that near-peer teachers learn and retain more than with passive learning [12, 13]. Active learning promotes practice using and interpreting concepts firsthand, allowing the learner more engagement with the material and revealing knowledge gaps for focused study [14]. Long-term memory of knowledge is also improved through experiential education [15]. 

With these concepts in mind, we present a novel, peer-led, experiential QI/PS curriculum for medical students that was implemented in a student-run free clinic setting to assess students’ perceptions in regard to (1) student confidence in QI methodology, and (2) competency in executing QI initiatives in healthcare.

## Methods

### Study design and duration

This was a quasi-experimental posttest-only study with one comparator group over nine months (July 2022-April 2023).

### Study setting

The University of Florida College of Medicine (UFCOM) is an allopathic medical school in Gainesville, Florida that has a four-year longitudinal QI/PS curriculum taught to all medical students throughout the preclinical and clinical years. Additional QI/PS training opportunities are available through the Patient Safety and Quality Discovery Track [16], an enhanced learning opportunity led by two clinical faculty members. This program offers faculty mentorship along with seminars focused on quality of care initiatives, analysis of adverse events, and has students complete the Institute for Healthcare Improvement (IHI) Open School Basic Certificate in Quality and Safety with meetings to discuss learning moments of each module [17]. 

The Equal Access Clinic Network (EACN) is the largest network of student-run free healthcare clinics in Florida [18]. Founded in 1988 by UFCOM students, student volunteers from the University of Florida’s undergraduate campus, pharmacy, physician associate, medical, dental, public health, clinical psychology, and physical therapy schools operate four primary care clinic sites including eight subspecialty clinics, with the support of University of Florida faculty members [18]. 

### Study participants

Twenty-three medical students, primarily made up of those in their second or third years of medical education, were recruited to build teams and implement a specific QI project at each of the four primary clinic sites. Two second-year medical students directed the EACN QI curriculum as QI project directors, working in close contact with the QI/PS faculty advisors, the EACN student director, and each clinic’s QI lead(s). Each clinic site had a team leader and 2–5 team members. Two clinic sites had first-year medical students to enable project continuity and sustainability.

### Experiential component

QI team leads and team members volunteered in their respective clinics to make observations regarding workflow, efficiency, and conducted root cause analyses to identify contributing factors that led to prolonged patient time in clinic. Brainstorming sessions were held with clinic directors, clinic officers, and volunteers to help identify interventions that could be tested and implemented using Plan-Do-Study-Act (PDSA) methodology to decrease the time patients spent in clinic without sacrificing patient care. Using stakeholder feedback collected in these brainstorming sessions, each of the four primary care clinics created site-specific key driver diagrams and swim-lane process maps for the workflow at their respective clinic. Recognizing the unique challenges the different clinic sites experienced and the importance of generating stakeholder buy-in, each team was given autonomy to conduct a minimum of three PDSA cycles based on discussions and partnership with each clinic site’s student directors.

**Fig. 1:**
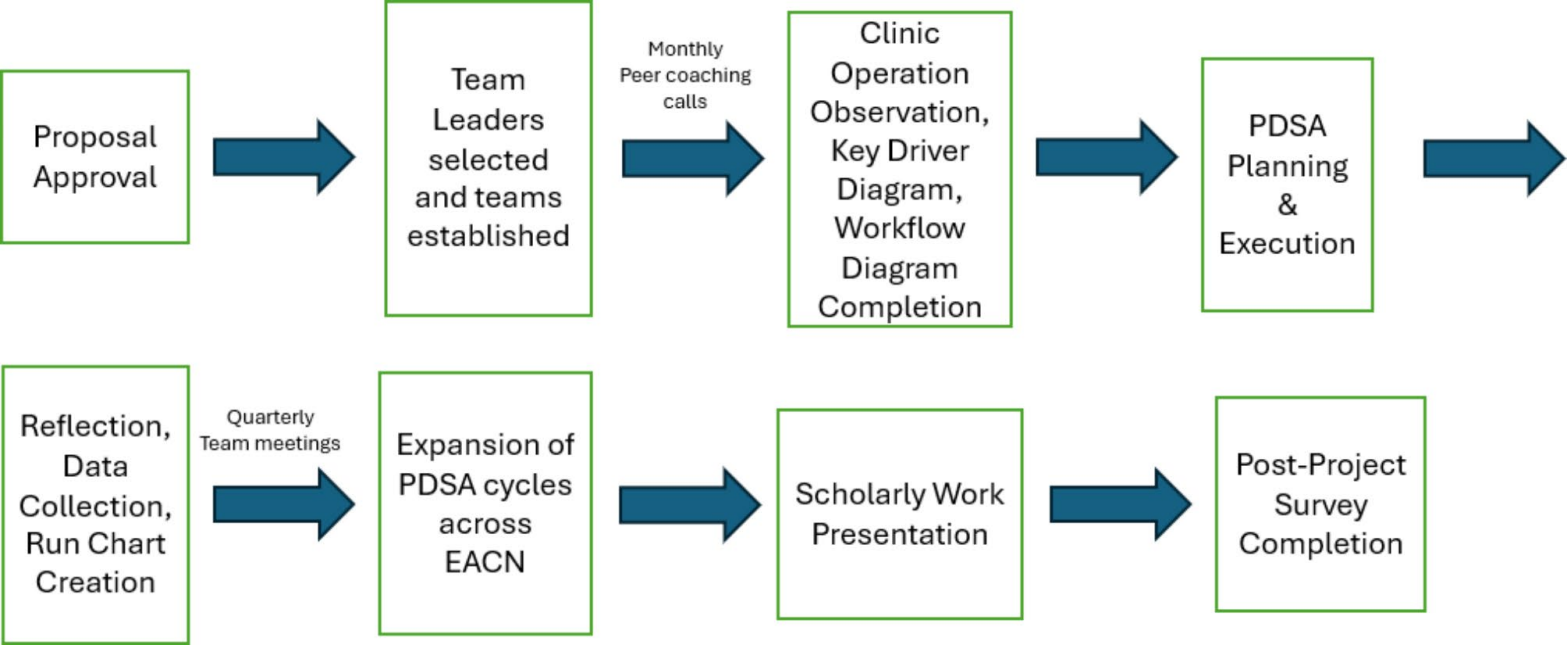
Diagram of experiential curriculum implementation

The QI/PS teams at each clinic planned and executed each PDSA cycle. Subjective data was collected using a PDSA journal/worksheet where QI teams reflected on challenges and ongoing questions during implementation. Objective data was collected by QI student project directors that measured each patient’s time in the clinic from arrival to departure. Patient time spent in clinic was plotted in clinic-specific and network-wide run charts and distributed to teams for tracking project impact and outcomes. Interventions were then adjusted and ultimately evaluated for network-wide implementation. Study implementation is described in Fig. 1.

### Peer-to-peer coaching

In addition to experiential learning, the leaders of each team received coaching from peer-/near-peer leaders and expert faculty members. Monthly check-ins between student QI project directors and QI leads were leveraged to update each clinic site on their patient cycle time progress, review QI tools/concepts, and discuss themes identified in the collected data. Quarterly, team leaders across all clinics met with peers and faculty to share key learnings and align network-wide initiatives.

### Optional institute for healthcare improvement open school

All participating students were encouraged to complete courses from the Institute for Healthcare Improvement’s online Open School curriculum and subsequently earn the Basic Certificate in Quality and Safety. This certification includes 13 virtual courses with an emphasis placed on the concepts of QI/PS, the Triple Aim for populations, patient- and family-centered care, and health care leadership [19]. 

### Optional supplementary modules

Optional, short, multimodal lessons on QI methodology were created by the student QI project directors and distributed via email on a monthly basis to the entire twenty-three student QI team in order to support achievement of the learning objectives. These topics included: (1) *Leadership and Gaining Buy-In from Stakeholders*; (2) *Development of a Specific*,* Measurable*,* Achievable*,* Relevant*,* and Time-bound (SMART) Goal*; (3) *Key Driver Diagrams*; (4) *Process Mapping*; (5) *Process Measures vs. Outcome Measures vs. Balancing Measures*; (6) *How to Interpret QI/PS Data*; (7) *How Quality Improvement Differs from Research*; (8) *Tips on QI/PS Presentation*; and (9) *Publishing a QI/PS Project*.

### Data analysis

A validated approach to measure the impact of educational training was developed by Douglas Kirkpatrick, who presented a model to evaluate training comprised of four levels: *Reaction*,* Learning*,* Behavior*,* and Results* [20–22]. To evaluate these key components, a voluntary, anonymous survey was distributed to all 23 student participants (*n* = 15 respondents) and to a control group of non-participating students across all four academic years at UFCOM (*n* = 43 respondents) via email. This survey included questions to assess overall perceptions of the QI initiative at EAC and incorporated the validated assessment tool *The Beliefs*,* Attitudes*,* Skills*,* and Confidence in Quality Improvement* (BASiC-QI) *Scale* used as a surrogate to measure the incremental knowledge and skills participants gained. The BASiC-QI Scale is positively correlated with the gold standard assessment tool, *Quality Improvement Knowledge Application Tool* (QIKAT-R), but leverages greater efficiency in its delivery [23]. Results were compiled and evaluated utilizing the The New World Kirkpatrick 4-level method and analysis of variance (ANOVA) was used to assess if there were differences in BASiC-QI scale response means between participants and non-participants of the QI project using R Statistical Software v4.2.2. Data was described in terms of mean (*M*) ± standard deviation (SD). A *p*-value less than 0.05 was considered statistically significant.

### Ethics approval and consent to participate

This research met criteria for exempt research and was authorized by UF Research Division of Research Operations under protocol # ET00019737, Implementation of a Quality Improvement Experiential Medical Curriculum.

## Results

This study included 15 of the 23 students who participated in the EAC QI project and 43 non-participating students. All respondents who completed this survey were included in the study and none of the students were excluded from the study. These students were medical students enrolled at UFCOM and included nine first-year students (16%), 31 second-year students (53%), 14 third-year students (24%), three fourth-year students (5%), and one student who declined sharing their year in school. Thirteen of the responding participants (87%) and five of the responding non-participants (12%) were concurrently enrolled in the UF Patient Safety and Quality Improvement Discovery Track.

The first level of Kirkpatrick training evaluation, *Reaction*, was evaluated using the BASiC-QI Scale attitudes questions to measure learners’ perceptions. Of the 15 respondents who participated in this QI project, 93% “strongly agreed” or “agreed” to the survey questions *“I enjoy QI*,*” “I am interested in QI*,*” “intend to participate in QI initiatives as a graduated healthcare professional*,*” “enjoyed being a part of the QI experiential training opportunity*,*” “believe applications of QI theory and methodologies can help make change to a system*,*”* and *“value QI training as part of their professional development.”* Additionally, 93% of participants believe *“there should be QI teams as part of EACN*,*”* and found *“value from having these teams integrated into the clinics*,*”* while 87% reflected that they learned more about QI from their participation in the project.

The second level of the Kirkpatrick training evaluation, *Learning*, involves measuring the knowledge and skills the participants gained and was assessed through the BASiC-QI Scale distributed to participants and a control group of non-participating medical students at the conclusion of the project. Participants scored significantly better than non-participants in all three sub-domains and total scores **(**Table 1).

**Table 1:** BASiC-QI scale sub-domain and total scores by project participation status

	Non-participants (*n* = 43) M (SD)	Participants (*n* = 15) M (SD)	*P*-value
Attitudes and Beliefs	52.00 (8.94)	58.53 (7.03)	0.013
Knowledge of QI	40.05 (10.85)	55.07 (5.61)	< 0.001
QI Skills	57.21 (11.39)	73.27 (9.09)	< 0.001
Total Score	149.26 (25.36)	186.87 (20.45)	< 0.001

There were no statistically significant relationships between student year, number of hours volunteered at EAC, or involvement/leadership within EAC when compared to the BASiC-QI Scale sub-domains and total scores. There was a non-significant increasing trend in knowledge of QI by school year (*p* = 0.076). No other trends by school year approached significance. (Tables 2, 3 and 4).

**Table 2:** BASiC-QI scale sub-domain and total scores by EACN volunteer hours

	0 h (*n* = 7) M (SD)	1–10 h (*n* = 11) M (SD)	11–20 h (*n* = 7) M (SD)	21–30 h (*n* = 8) M (SD)	31–40 h (*n* = 11) M (SD)	41 + Hours (*n* = 14) M (SD)	*P*-value
Attitudes and Beliefs	56.14 (6.18)	54.18 (5.40)	51.43 (7.89)	52.62 (14.26)	53.36 (10.13)	54.07 (9.14)	0.953
Knowledge of QI	46.71 (11.21)	43.36 (12.89)	45.57 (9.69)	35.38 (11.43)	45.73 (13.21)	45.64 (10.81)	0.386
QI Skills	64.14 (13.31)	61.09 (11.90)	60.86 (8.63)	54.25 (8.71)	67.18 (11.65)	59.93 (17.08)	0.403
Total Score	167.00 (28.86)	158.64 (27.58)	157.86 (21.04)	142.25 (25.81)	166.27 (33.09)	159.64 (33.31)	0.586

**Table 3:** BASiC-QI Scale sub-domain and total scores by EACN leadership status

	Non-leadership (*n* = 33) M (SD)	Leadership (*n* = 25) M (SD)	*P*-value
Attitudes and Beliefs	52.55 (9.11)	55.20 (8.59)	0.265
Knowledge of QI	42.94 (11.77)	45.24 (11.89)	0.466
QI Skills	60.61 (11.63)	62.36 (14.57)	0.612
Total Score	156.09 (27.98)	162.80 (30.89)	0.391

**Table 4:** BASiC-QI Scale sub-domain and total scores by student year

	MS1 (*n* = 9) M (SD)	MS2 (*n* = 31) M (SD)	MS3 (*n* = 14) M (SD)	MS4 (*n* = 3) M (SD)	*P*-value
Attitudes and Beliefs	56.56 (6.67)	53.74 (8.52)	52.21 (10.15)	48.33 (13.65)	0.507
Knowledge of QI	34.78 (17.21)	44.39 (10.17)	46.43 (8.20)	48.67 (11.59)	0.076
QI Skills	58.33 (13.18)	60.58 (13.52)	62.86 (11.33)	64.00 (11.53)	0.832
Total Score	149.67 (33.76)	158.71 (29.15)	161.50 (24.65)	161.00 (36.01)	0.805

The third level of the Kirkpatrick model to evaluate training is *Behavior*, reflecting an assessment of the learners’ ability to apply their new knowledge and/or skills through hands-on use of the techniques and tools. A total of 18 PDSA cycles were completed during this project, averaging 4.25 per QI team. Scholarly activity produced by project participants included six poster presentations and two oral presentations at national/international conferences, along with nine poster presentations and three oral presentations at local/regional venues. Five manuscripts associated with this project have been submitted for publication, with four accepted for publication at the writing of this manuscript [24, 25].

**Fig. 2:**
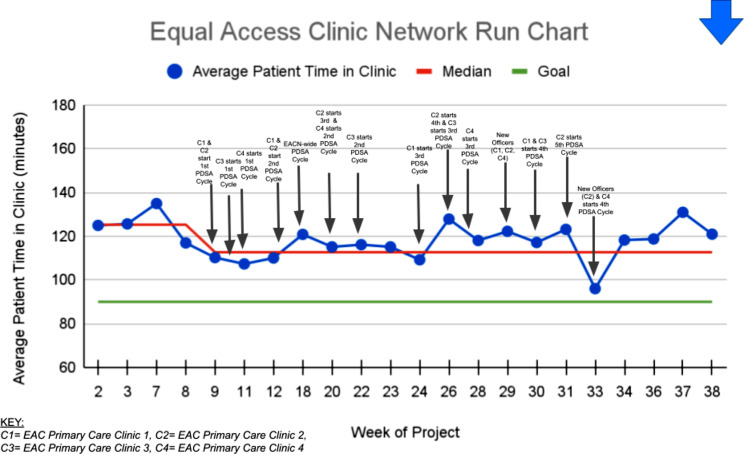
Equal Access Clinic Network Run Chart, excluding weeks that did not have data from all four clinics (weeks 1, 4, 5, 6, 10, 13, 14, 15, 16, 17, 19, 21)

The fourth Kirkpatrick level of training evaluation is *Results*, or a measure of the impact that the training has overall. In this case, the median time patients spent in clinic across the EACN decreased by 12.6 min or 10.1% (125.3 min to 112.7 min) over the duration of this project, indicating a significant run chart shift (Fig. 2).

## Discussion

This project successfully established a platform for hands-on QI education, allowing medical student participants to execute and present a QI project, identify and gain stakeholder buy-in, learn to apply QI tools in practice, and how to systematically improve patient care. There is an increased focus on incorporating QI/PS curriculum into medical education. With approximately 75% of AAMC-accredited medical schools operating student-run free clinics, this project demonstrates an opportunity for these clinics to provide medical students applied QI/PS experience in a real-world context [10]. 

Based on the The New World Kirkpatrick 4-level method of evaluating training programs, this experiential quality improvement curriculum resulted in positive outcomes in all four levels: *Reaction*,* Learning*,* Behaviors*,* and Results.* It is noted by *Brown*, et al. that, “fostering positive attitudes and behaviors should be considered an important aspect of any QI curriculum…as poor attitudes towards QI could result in resistance to improvement efforts in the health system.” [23] Therefore, we want to highlight that 93% of the EAC QI project participants answered they *“enjoyed being a part of the QI experiential training opportunity*,” *“believe applications of QI theory and methodologies can help make change to a system*,*”* and *“value QI training as part of their professional development.”*

The EAC QI project participants also demonstrated a statistically significant increase in QI knowledge, confidence, and skills compared to non-participants based on their responses on the BASiC-QI Scale. Meanwhile, teams exhibited their work across numerous poster presentations, oral presentations, and manuscripts, demonstrating participants’ successful utilization and application of the learning content and ability to leverage QI tools–such as key driver diagrams, process maps, and run charts–to design and conduct a QI project and share results with the broader medical community. Finally, students were able to realize their impact on patient care through a significant 10.1% reduction in patient cycle time. Generally, a shift of six or more data points indicates a change attributable to the intervention(s). Given no comparator was evaluated due to the nature of the project, the possibility exists that this reduction in patient cycle time could be attributed to an unmeasured or unknown contributing factor.

There was no statistically significant relationship between student year, number of hours volunteered, or involvement/leadership in EAC and the BASiC-QI Scale sub-domains and total scores. There was a non-significant increasing trend in knowledge of QI by school year (*p* = 0.076). No other trends by school year approached significance. Overall, this indicates that the higher value of scores for participants was due to their involvement in the project and not merely general increased clinical exposure. However, another unmeasured factor could have contributed to the significant difference between groups. Such unmeasured factors could include project participants having a background in QI, an increased interest in QI, QI experience, or QI independent study prior to or during the project.

Peer- and near-peer teaching are expected to enhance the retention of concepts, equipping medical student participants to confidently engage in process improvement as residents and future physicians. While peer-/near-peer teaching has its strengths, some may consider this a limitation of this curriculum. One may anticipate that teaching/learning opportunities may have been weakened, key concepts de-emphasized, or there could have been introduction of bias within a peer-driven educational platform. Although faculty did not directly teach the information, the student QI project directors had significant faculty oversight. Educational outcomes and learning objectives were established with faculty partnership (**Appendix**). Concepts, QI tools, presentations, and publications were reviewed by faculty mentors throughout the project.

Evaluation of outcomes using surveys, as in this study, has limitations. These include low participation potentially introducing non-response bias to the results or providing answers deemed desirable by the surveyor introducing response bias. In this case, the surveys remained anonymous. Utilization of a validated assessment, the BASiC-QI Scale, helped to mitigate the risk of misinterpretation of survey questions as it has been standardized and rigorously tested [23]. 

A limitation of our research is that the BASiC-QI Scale has not been established as a tool to compare against a control group. Few tools exist in the healthcare literature that adequately, objectively, and reliably assess QI-related competencies. The gold-standard QIKAT-R requires the use of multiple trained raters in addition to a significant time investment to evaluate learners’ QI knowledge. The BASiC-QI Scale has been validated against the gold-standard of QIKAT-R to evaluate changes over time in learners’ beliefs, attitudes, skills and competencies throughout QI training [23]. Therefore, we believe the BASiC-QI Scale is an adequate measure to indicate overall QI confidence and approximation of gained QI skills. Future reliability testing amongst comparison groups is necessary to better understand the significance of using the BASiC-QI Scale for comparison of QI knowledge between QI project participants and a control group.

The medical education component of this project aims to ensure continuity, with the goal of sustaining medical student engagement in QI initiatives so as to build confidence and knowledge in QI practices. This will generate new ideas and process improvements that promote an enduring commitment to practicing high value care at our student-run free clinic network. Next steps include incorporation of QI teams into the EACN on a permanent basis, along with continued partnership of the Patient Safety & Quality Discovery Track. This will allow new teams of students to continue QI initiatives, now in its third year of implementation. Considerations for future iterations of this project include utilizing a formalized didactic curriculum and making the IHI Open School Basic Certificate in Quality and Safety completion a prerequisite for QI project participation in order to foster increased engagement and QI proficiency.

Overall, the presence of a student-led QI team in the EACN resulted in a decrease in the time patients spent in our clinics across the student-run free clinic network. Furthermore, medical students received relevant, tailored QI education and utilized newly acquired QI skills in a real-world context. Therefore, implementation of an experiential QI/PS curriculum in student-run clinics allows medical schools to achieve benchmark goals of the AAMC, prepare students to fulfill future ACGME residency requirements and later career opportunities, and create significant impact on the future healthcare landscape and patient experience.

## Conclusions

Quality improvement remains a key focus in medical education, emphasized by both the AAMC and ACGME. Optimistically, early exposure to hands-on QI tools and methodologies can equip medical students to strategically address future challenges that could allow them to implement data-driven changes in their future practice. Incorporating a student-led QI team into student-run free clinics fosters this experience, creating a high value care ripple effect that extends far beyond the scope of any one project.

## Appendix

### Experiential curriculum objectives

Appropriate learning objectives and educational outcomes **(**Table 5) were informed by IHI education platforms and literature review, and established through faculty partnership with Project Directors [19, 26].

**Table 5:** Educational outcomes and learning objectives associated with an experiential QI project implemented into medical education [19, 26].

Educational Objectives	Learning Objectives
Teach collaborative skills	● Explain several tactics for strengthening communication and teamwork to help make a clinical improvement project successful in the real world
Provide learners with opportunities to work with colleagues from other disciplines	● Explain the importance of having support from peers and colleagues in health care ● Establish comfort level in engaging stakeholders at different levels of an organization’s hierarchy to address systems problems
Provide intensive coaching from expert faculty members	● List some of the differences between measurement for improvement and measurement for academic research
Provide students with access to their own performance data	● Identify three kinds of measures: process measures, outcome measures, and balancing measures ● Explain the value of tracking and plotting data over time ● List the basic elements of an effective run chart ● Apply four rules to identify non-random variation in the data on a run chart ● Identify a shift in a run chart ● Explain the basics of displaying data for improvement
Facilitate experiential learning with incremental change from trial and error	● Identify the processes that make up the Plan-Do-Study-Act (PDSA) cycle ● Explain the importance in improvement work of conducting iterative small-scale tests of change ● Use a PDSA worksheet to plan and conduct a small test of change for your own personal improvement project ● Establish comfort level to: o Plan a PDSA cycle o Design a PDSA cycle o Implement a PDSA cycle
Provide trainees with access to process improvement tools, to enable them to focus on implementing interventions rather than developing their own tools	● Explain how visual tools, such as driver diagrams, can help at different phases of improvement ● Use the Model for Improvement to begin your own personal improvement project ● Explain the importance of gathering objective data about a problem and seeking solutions ● State why it’s important to set an aim statement at the start of an improvement project ● Describe how to strategically adjust the size and scope of PDSA test cycles over time ● Describe the phases of an improvement project ● Rate your comfort level in utilizing and creating process improvement tools

## Data Availability

No datasets were generated or analysed during the current study.

## Abbreviations

QI/PS: Quality improvement and patient safety
QI: Quality improvement
ACGME: Accreditation Council for Graduate Medical Education
CanMEDS: Canadian Medical Education Directive for Specialists
AAMC: Association of American Medical Colleges
UFCOM: University of Florida College of Medicine
UF: University of Florida
EACN: Equal Access Clinic Network
IHI: Institute for Healthcare Improvement
PDSA: Plan-Do-Study-Act
BASiC-QI: Beliefs, Attitudes, Skills, and Confidence in Quality Improvement
QIKAT-R: Quality Improvement Knowledge Application Tool
ANOVA: Analysis of variance
